# Time trends: a ten-year comparison (2005–2015) of pedometer-determined physical activity and obesity in Czech preschool children

**DOI:** 10.1186/s12889-016-3269-5

**Published:** 2016-07-13

**Authors:** Erik Sigmund, Dagmar Sigmundová, Petr Badura, Lucie Trhlíková, Andrea Madarasová Gecková

**Affiliations:** Institute of Active Lifestyle, Faculty of Physical Culture, Palacky University Olomouc, Olomouc, Czech Republic; Department of Health Psychology, Faculty of Medicine, Safarik University, Kosice, Slovakia; Graduate School Kosice Institute for Society and Health, Safarik University, Kosice, Slovakia

**Keywords:** Step count, Yamax pedometer, Trend, Weekdays, Weekend, Kindergarten, Leisure-time, Obesity

## Abstract

**Background:**

To explore the time trends (2005–2015) of pedometer-determined weekday and weekend physical activity (PA) and obesity prevalence in 4–7-year-old Czech preschool children and changes in proportion of kindergarten vs. leisure-time PA.

**Methods:**

The study compared data of two cross-sectional cohorts of preschool children (2005: 92 boys and 84 girls; 2015: 105 boys and 87 girls) in the Czech Republic, using the same measurements and procedures in both cases. PA was monitored by the Yamax Digiwalker SW-200 pedometer for at least eight continuous hours a day over seven consecutive days. Body weight and height were measured using calibrated Tanita scales and anthropometry. The analysis of variance was conducted to examine the gender and cohort effect on step counts. The *t*-test was used to examine the difference in step counts in kindergarten (or leisure-time) between non-obese and obese children, and the chi-square test compared the prevalence of obesity between 2005 and 2015.

**Results:**

The steps/day (mean ± standard deviation) of preschoolers was significantly higher (*p* < 0.05) in 2015 (11,739 ± 4,229 steps/day) than in 2005 (10,922 ± 3,181 steps/day); and (*p* < 0.001) in boys (11,939 ± 3,855 steps/day) than in girls (10,668 ± 3,587 steps/day). In 2015, girls, but not boys, had a significantly (*p* < 0.01) greater step count on weekdays than in 2005, but not at weekends. A decline of leisure-time step counts on weekdays between 2005 and 2015 in girls (6,865_2005_ vs. 6,059_2015_, *p* < 0.01) and boys (7,861_2005_ vs. 6,436_2015_, *p* < 0.001) is compensated for by the increase of step counts in kindergarten (girls: 3,058_2005_ vs. 5,330_2015_, and boys: 4,003_2005_ vs. 5,999_2015_, *p* < 0.001). The prevalence of obesity was not significantly different either in 2005 or 2015 among preschool girls (7.14 % _2005_ vs. 9.20 % _2015_) or boys (6.52 % _2005_ vs. 9.52 % _2015_).

**Conclusion:**

The steps/day of preschoolers was higher in 2015 than in 2005; this higher level of PA was the result of increased PA in kindergartens over the last ten years, particularly among girls. Thus, the current PA program in kindergartens effectively compensates for the decline in PA in leisure-time of weekdays of non-obese and obese preschoolers compared to 2005 and 2015. Prevalence of obesity among Czech preschool children remains relatively stable between 2005 and 2015.

## Background

Risk for obesity in adolescence [1, 2] and adulthood [3, 4] starts in early childhood, as well as in early childhood starts to develop a physically active lifestyle in adulthood [5, 6]. In the last 15 years, however, in the context of a technology-driven increase in sedentary lifestyle and, at the same time, a decline in physical activity (PA) [7, 8], obesity prevalence in school-aged children and adolescents has been rising [9] or reaching a plateau, which remains historically high [10, 11]. The finding that the peak prevalence of obesity in developing countries has moved from older adults to younger age cohorts [12] is very disturbing. Will the increase in overweight and obesity and the decline of PA also be apparent in preschool children?

Despite the diversity of study designs and measurement methodologies, cross-sectional data from 144 countries between 1990 and 2010 consistently indicate a dramatic increase of prevalence of overweight/obesity of preschool children in developed or developing countries [13]. The relative percentage change of prevalence of overweight/obesity shown an increase of 48 % in developed or 65 % in developing countries between 1990 and 2010 [11]. However, more recent studies have revealed a different pattern of trend in the overweight/obesity prevalence in preschool children [9, 14]. While in English children aged 2–5 years an increase in the prevalence of overweight/obesity was found (boys: 21.4 %-25.2 %; girls: 19.4 %-22.3 %) between 1994–2003, this increasing trend stopped in the following period (2004–2013) and the prevalence of obesity stabilised (boys: 24.9 %; girls: 23.8 %) [9]. Similarly, the prevalence of obesity among Chinese children aged 5–6 years increased from 8.8 % to 10.1 % between 2006–2010, and was then stable until 2014 (10.1 %) [14]. A persistent state in the prevalence of obesity was observed also in 4–5-year-old New Zealand children between 2009 and 2012 (boys: 20.1 %-19.2 %; girls: 17.5 %-16.6 %) [15]. In a retrospective study of preschool children in the Czech Republic, which covered the 1957–2015 period, the authors found a significant increase in trunk fat mass and a simultaneous decrease in motor performance, but PA was not monitored [16].

In terms of objectively pedometer monitored day-long PA of preschoolers, previous studies [17–19] have documented that preschool children are more physically active than first-grade [17, 18] and second-to-third-grade school children [18] and adolescents aged 12–19 years [19]. In addition, several European studies repeatedly revealed higher step counts on weekdays than at weekends [20–22] and no differences in step counts per day between boys and girls [20, 23]. However, among Czech preschool children were found no differences in step counts per day either between boys and girls, or between weekdays and weekend days [17].

Globally, there is a lack of published data on the time trends of obesity in preschool children [10, 24, 25], as well as on their time trends of objectively monitored PA [26], especially in the countries of Central and Eastern Europe [17, 27]. Although the objective monitoring of PA in preschool children is occasionally implemented in the countries of Western and Northern Europe [28, 29], the countries of Central and Eastern Europe are rarely included in such studies [21, 30]. In addition, analysis of objectively monitored PA in kindergarten, and during the leisure-time of preschool children, is still insufficiently explored. To the best of our knowledge, this is the first study from the Central and Eastern European countries describing trends in pedometer-determined PA and obesity in preschoolers. The present study captures trends (2005–2015) in objectively monitored PA (daily step counts – employing pedometers) and obesity in Czech preschool children.

The primary aim of this study was to explore time trends of pedometer-determined weekday and weekend step counts, and the prevalence of obese preschool children, from 2005 to 2015. In addition, we examined changes in proportion of kindergarten vs. leisure-time step counts on weekdays in 2005 and 2015 in obese and non-obese children, and the percentage of preschool children achieving PA guidelines expressed in steps/day.

## Methods

### Subjects

#### Sample

This study compared two cross-sectional cohorts from 2005 and 2015. Out of the 22 stratified selected public kindergartens in the Moravian region of the Czech Republic in 2005, 11 agreed to participate in the survey after a detailed presentation of the study [17]. Stratified sampling selection included public kindergarten from all sub-regions of the Moravian region and included kindergartens from cities and rural areas [31, 32]. All selected public kindergarten have its own garden (with an outdoor playground), and are located in a separate building [17, 27]. During April/May and September/October 2005 under comparable daily climate, data of pedometer-determined PA and Body Mass Index (BMI) were collected from 176 preschool children (84 girls) from an initial number of 208 (106 girls) aged 4–7 years, in 11 public kindergartens in the Moravian region of the Czech Republic [17].

During April/May and September/October 2015, 228 preschool children aged 4–7 years, from the ten public kindergartens in the same settlement units as in 2005, were asked to participate. Eight out of the ten kindergartens that agreed to participate in the 2015 survey had also participated in 2005. The remaining two kindergartens selected for survey in 2015 were chosen from nearby kindergartens. All the participating kindergartens in 2015 met the same conditions as those in 2005. Two hundred and ten (96 girls) children were included, out of which 192 children (87 girls) completed the study. Reasons for exclusion of participants in both cohorts in the cross-sectional comparative study were as follows: outbreak of disease in the course of research; failure to complete the entire monitoring; or incomplete data of daily step counts. Participating preschool children of both cohorts were predominantly white Caucasian (>95 %), which is representative of the ethnic demographics in this part of the Czech Republic [31].

#### Kindergartens

All the participating public kindergartens were located in separate houses surrounded by a grassy garden, with a children’s sandpit and an age-appropriate children’s playground. Each of the participating kindergartens in 2005 and 2015 provided a daily 30–60 min walk outdoors, and 20 min of indoor exercise steps and dance variations, competitive and coordination movement games, relaxation or breathing activities, and other types of exercise. This PA programme is standard for all public kindergartens in the Czech Republic [17, 32]. Other PA beyond the common daily programme could be provided during the time spent outdoors in the garden under the supervision of teacher or during extra parents paid lessons (eg. the basics of swimming, athletics, inline skating) [32]. Czech teachers in public kindergartens are university-educated teachers specializing in versatile preschool education, which includes development of motor skills [32].

### Measurements

#### Physical activity monitoring

Data were collected following the same design and identical procedures in 2005 and 2015 under the leadership of the same researchers. The same type of pedometer – the Yamax Digiwalker SW-200 (Yamax Corporation, Tokyo, Japan) and identical personalised individual logbooks were used for PA monitoring. The study participants were instructed to record their PA for whole day over eight days [17, 32] to be included in the analyses.

The Yamax Digiwalker SW-200 is a non-expensive, small and light electronic pedometer. Via a pendulum arm moving with the vertical oscillations of walking its circuit switches on and off [33]. Each vertical oscillation that exceeds the device threshold (#0.35 g) counts as a step [34]. The total step count, which is the most accurate pedometer-derived variable representing PA [35], is shown on the display of the device. Pedometer-based step counts are valid estimates of preschoolers’ physical activity levels during free-living activities, based on group estimates in comparison with accelerometer-based steps (hourly: *r* = 0.92; daily: *r* = 0.89) [36] or in comparison with direct observation (R^2^ = 0.59) [37].

The personalised individual logbook consisted of two parts that were filled in by parents/teachers: one for providing the anonymised code of the participating child, the start and end dates of monitoring PA, and the code of the kindergarten. The other section was intended for recording the step counts and included the chronological structure of the day to note the time and value shown on the display (step count) of the Yamax pedometer four times a day (morning after waking up – by parent; start and end of kindergarten – by teacher; evening before going to bed – by parent) [17, 32].

At the beginning of the eight-day PA monitoring period, children were given an elastic belt with a pocket for the Yamax pedometer and a personalised individual logbook. The Yamax pedometer was not to be reset in the course of the day. The parents and teachers were instructed to ensure that children wore the pedometer on their right hip, for at least eight hours a day, except during personal hygiene, bathing, rest time, and sleeping, [17, 32]. The parents and teachers were trained to check the correct attachment of the pedometer, and enter correctly the pedometer data into the personalised individual logbook. Using the interval between the morning (pedometer turned on) and evening (pedometer turned off) we computed daily wearing time. Data analysis did not include the first monitoring day since the data on the first day was incomplete and the novelty of wearing the pedometer could have affected the initial PA (reactivity) [38]. The data analysis included only records when the pedometer was worn for at least eight hours a day on at least four weekdays and both weekend days [39]. Monitoring of at least four weekdays and two weekend days was appropriate for predicting weekly PA in children and for comparison of pedometer-based step counts between weekdays and weekend days [32, 39].

#### Anthropometry

In advance before the PA monitoring itself the anthropometric measurements of the participating children were carried out in order to prepare an individual logbook for each child. The week before the start of PA monitoring, participants were given the opportunity to familiarise themselves with the Yamax pedometers during a joint meeting at each of the participating kindergartens. At this time, measures of height and weight were collected from all the participants to determine their BMI. Height was measured using the A-319 Anthropometer (Trystom Corporation, Olomouc, Czech Republic) and rounded to the nearest centimetre. Weight was measured using the calibrated Tanita WB 110 S MA (Quick Medical Corporation, Seattle, WA, USA) and rounded to the nearest 0.5 kg. The chronological age was calculated from the date of birth until the first day of PA monitoring. Age-specific cut-off points, according to the World Health Organization [40], were used to define the prevalence of obesity.

### Data analysis

Data were analysed using SPSS v22 software (IBM SPSS, Inc., Chicago, IL, USA) and STATISTICA v.12 (StatSoft, Prague, Czech Republic). The BMI was computed as the body weight (kg) divided by body height (m) squared. Obesity in children was classified using the WHO percentile BMI charts for girls and boys between the ages of 5 and 19, where obesity was represented by >97 % on age-differentiated BMI charts [40]. To determine obesity in children aged less than five, we used corresponding age-differentiated BMI charts [41]. The step counts data of the seven days was reviewed to check for missing and extreme values. If step counts were recorded during four weekdays, data for the missing one weekday were added, based on the participant’s personal mean scores (in the 2005 cohort: 5.1 %, in 2015 cohort: 5.8 %). Participants (*n* = 5; n_2005_ = 3 and n_2015_ = 2) whose step count data were missing for more than one day were excluded from analysis. In the case of 15 children (n_2005_ = 5 and n_2015_ = 10), daily step counts under 1,000 or exceeding 30,000 in one of monitored day were truncated to 1,000 or 30,000 [19, 39], and were included in the final analysis. The daily step count on weekdays comprised the sum of the kindergarten step count and leisure-time step count. A kindergarten step count indicated the number of steps in kindergarten (from morning arrival in kindergarten to afternoon departure). A leisure-time step count represented the sum of the number of steps before the start of kindergarten and the number following departure from kindergarten in the evening on weekdays. The variable of daily step count at weekends represented the mean difference between morning (pedometer turned on) and evening (pedometer turned off) step counts on Saturday and Sunday. The assessment of achieving PA recommendations was determined according to the Czech pedometer-based step count guidelines for preschool children of 11,500 steps per day [32], which is consistent with published step counts target of 11,500 steps per day as an equivalent of 180 min of total PA per day [22].

The data were analysed in total for two cohorts of preschool children because the TwoStep cluster analysis yielded no indication for clustering by kindergarten or season in 2005 or 2015. Gender-stratified means, standard deviation (SD), and 95 % confidence intervals (CI) were calculated for step counts for whole week day, weekdays, weekends, kindergarten time of weekdays, and leisure-time of weekdays. Two two-way analysis of variance (ANOVA) for repeated measures were computed to assess the gender and cohort effect on overall weekdays/weekend daily step counts, and kindergarten/leisure-time step counts. Subsequently, to identify the differences in step counts between weekdays (kindergarten and leisure-time) and weekends in boys and girls, the Fisher’s LSD post-hoc test was used. Before using ANOVA for repeated measures normality of data distribution, homogeneity of variances, and of outliers of variables weekdays, weekends, kindergarten and leisure-time step counts were verified. It also verified condition of sphericity. The estimate of the strength of the relationships between the independent variables (affiliation with a cohort, gender) and step counts variable on weekdays, weekends, kindergarten and leisure-time of weekdays was represented by Cohen’s d effect size coefficient [42]. The values of 0.2, 0.5 and 0.8 were interpreted as small, medium and large effect sizes, respectively [43, 44]. To examine the cohort differences in step counts in kindergarten (or leisure-time) between non-obese and obese children, the *t*-test was used. The chi-square test compared the prevalence of obesity as well as the prevalence of those children, who met the recommended daily step counts by gender and cohort.

## Results

In total, data from 197 preschool boys and 171 girls aged 4–7 years were eligible for analysis. The means, standard deviations and relative frequencies of the children’s anthropometric characteristics and PA data are presented in Table 1.

**Table 1:** Anthropometric characteristics and pedometer-determined physical activity of two cohorts of 4- and 7-years-old preschool children

	2005				2015			
	Boys		Girls		Boys		Girls	
	(*n* = 92)		(*n* = 84)		(*n* = 105)		(*n* = 87)	
	M	(SD)	M	(SD)	M	(SD)	M	(SD)
Anthropometric data								
Age [years]	5.58	(0.46)	5.70	(0.82)	5.65	(0.82)	5.56	(0.81)
Body height [cm]	119.17	(7.18)	116.71	(4.30)	117.70	(7.43)	115.55	(7.95)
Body weight [kg]	22.26	(2.89)	21.12	(2.95)	21.46	(3.81)	20.41	(3.88)
Body Mass Index [kg/m^2^]	15.65	(1.38)	15.47	(1.65)	15.43	(1.88)	15.19	(2.44)
Obesity	*n* = 6 (6.52 %)		*n* = 6 (7.14 %)		*n* = 10 (9.52 %)		*n* = 8 (9.20 %)	
PA data - step counts								
Whole week day [mean]	11523	(3161)	10264	(3071)	12303	(4341)	11057	(3984)
Weekdays [mean]	11864	(2588)	9923	(2431)	12435	(4278)	11389	(3506)
Weekends [mean]	11182	(3643)	10606	(3597)	12172	(4441)	10725	(4425)
Kindergarten [mean]	4003	(1449)	3058	(1207)	5999	(2421)	5330	(2012)
Leisure time [mean]	7861	(2230)	6865	(1708)	6436	(3519)	6059	(2557)

*n* number of participants, *M* mean, *SD* standard deviation, *%* percentage, *PA* physical activity

Two-way ANOVA for repeated measures determined the significant effect of cohort (F = 5.26, *p* < 0.05, d = 0.22) and gender (F = 13.33, *p* < 0.001, d = 0.34) on the overall daily step count of preschool children. The daily step count (mean ± standard deviation) was significantly higher (*p* < 0.05) in preschool children in 2015 (11,739 ± 4,229 steps/day) than in 2005 (10,922 ± 3,181 steps/day) and overall (*p* < 0.001) in boys (11,939 ± 3,855 steps/day) than girls (10,668 ± 3,587 steps/day).

**Fig. 1:**
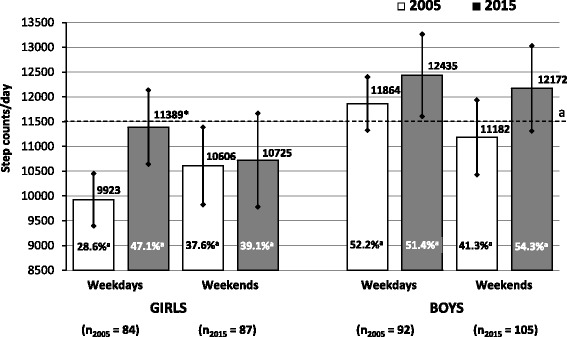
Pedometer-determined daily step counts of preschool children in 2005 and 2015. Mean and 95 % confidence intervals are presented separately for girls and boys on weekdays and weekend, n – number of participants in each cohort, a – the horizontal line represents recommendation 11,500 steps/day [22, 32], %^a^ – percentages of children who reach step counts recommendation [22, 32]. The statistical significance (two-way ANOVA for repeated measures, Fisher’s LSD post-hoc test) of the differences between 2005 and 2015 weekday (weekend) step counts is expressed as *p < 0.01

In addition, as regards the significant effect of cohort and gender, there was a significant interaction between the cohort, days of week (week day vs. weekend), and gender on step count (F = 5.75, *p* < 0.01). In weekdays, girls had a significantly (*p* < 0.01, d = 0.47) greater amount of steps (+1,466 steps/day) in 2015 than in 2005 but not at weekends (Fig. 1). In the same period, we found a small non-significant increase in step counts among boys on weekdays and also at weekends.

Following the cross-cohort analysis of daily step count, significant differences in achievement step counts based PA guidelines (11,500 steps/day [22, 32]) assessed by the chi-square test were revealed in preschool children between weekdays and weekends and also between 2005 and 2015. In detail, in the prevalence of meeting PA guidelines on weekdays boys achieved significantly higher values than girls (51.78 % _BOYS_ vs. 38.01 % _GIRLS_*p* < 0.01). The girls, on weekdays 2015, reached a significantly higher prevalence of meeting PA guidelines than in 2005 (47.13 % _2015_ vs. 28.57 % _2005_*p* < 0.01).

Less than 10 % were classified as obese, and we do not confirmed gender difference in obesity prevalence neither in 2005 nor in 2015. The prevalence of obesity was not significantly different either in 2005 vs 2015 among preschool girls (7.14 % _2005_ vs. 9.20 % _2015_) or boys (6.52 % _2005_ vs. 9.52 % _2015_).

**Fig. 2:**
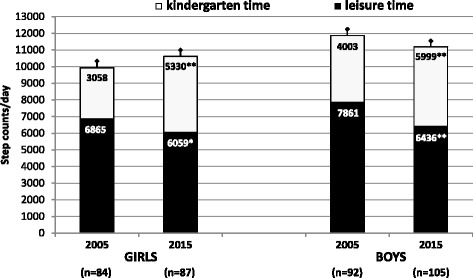
Pedometer-determined kindergarten and leisure-time step counts of preschool children on weekdays in 2005 and 2015. Mean and 95 % confidence intervals are presented separately for girls and boys, n – number of participants. The statistical significance (two-way ANOVA, Fisher’s LSD post-hoc test) of the differences between 2005 and 2015 kindergarten (leisure) time step counts is expressed as *p < 0.01 and **p < 0.001

Two-way ANOVA for repeated measures revealed the significant effect of the part of the day (kindergarten/leisure-time) (F = 179.06, p < 0.001, d = 0.86), and interaction effect of part of the day and cohort (F = 96.99, *p* < 0.001, d_KINDERGARTEN_ = 1.13, d_LEISURE TIME_ = 0.42) on the daily step counts on weekdays (sum of kindergarten step count and leisure-time step count). When comparing 2005 and 2015, we found a significant increase in kindergarten step count (girls: +2,272 steps/kindergarten time, *p* < 0.001, d = 1.37; boys: +1,966 steps/kindergarten time, *p* < 0.001, d = 0.99) among preschoolers of both genders (Fig. 2). At the same time, however, a significant decrease in the leisure-time step count in girls (−806 steps/leisure-time, *p* < 0.01, d = 0.37) and boys (−1,425 steps/leisure-time, *p* < 0.001, d = 0.48) was observed (Fig. 2). In connection with the significant change of both the kindergarten and leisure-time step count between 2005 and 2015, the ratio of the kindergarten (leisure-time) step count to the total daily step count also fundamentally changed. In 2005, preschool children achieved about twice as much steps in leisure-time than in kindergarten (*p* < 0.001); however, in 2015 this significant difference almost disappeared (Fig. 2).

**Fig. 3:**
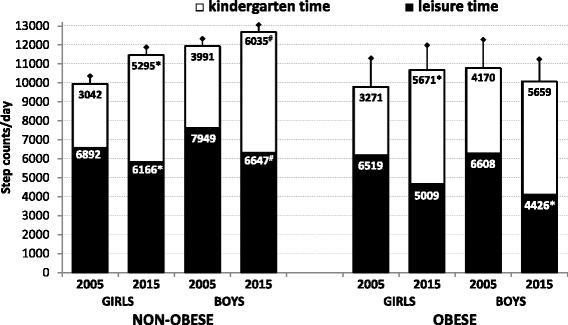
The proportion of kindergarten and leisure-time step counts in non-obese and obese boys and girls. Mean and 95 % confidence intervals are presented separately for non-obese and obese girls and boys. The statistical significance (*t*-test) of the differences between 2005 and 2015 kindergarten (leisure) time step counts is expressed as *p < 0.05 and ^#^ *p* < 0.005

In 2015, using the *t*-test, we found a significant increase in the kindergarten step count in obese girls (+2,400 steps/kindergarten time, *p* < 0.05, d = 3.39) and a significant decrease in leisure-time step count in obese boys (−2,182 steps/leisure-time, *p* < 0.05, d = 2.35) compared with 2005. There was no significant difference in kindergarten step counts between obese and non-obese girls, or obese and non-obese boys, either in 2005 or 2015. Similarly, we found no significant difference in the leisure-time step count between obese and non-obese girls, or obese and non-obese boys in 2005, as in 2015 (Fig. 3).

## Discussion

The trends of objectively monitored or self-reported PA and obesity in adolescents and young adults have been extensively reported [9–12, 45–47], but markedly less attention has been paid to these trends in preschool children [9, 13–15]. The present study extends understanding in this area by identifying time trends in weekday (kindergarten and leisure-time) and weekend pedometer-based step counts, and the prevalence of obesity in preschool-aged children. A key finding of this study lies in the discovery of a significant increase in pedometer-determined daily step counts in boys and girls between 2005 and 2015, and a slight, non-significant increase in the prevalence of obesity in the same period (boys: 6.52 % _2005_–9.52 % _2015_, +3 %; girls: 7.14 % _2005_–9.20 % _2015_, +2.6 %). The higher level of PA in 2015 than in 2005 is an effect of the increased PA in kindergartens. Since 2010, the Czech government supports projects for improving education of kindergarten teachers as well as projects focusing on healthy eating, material facilities and PA equipment of public kindergartens [32]. Thus, the current PA program in kindergartens effectively compensates for the decline in PA in leisure-time of weekdays, and even helps to increase the week-long PA of non-obese and obese preschoolers of both genders compared to 2005 and 2015.

The beneficial impact of regular PA on preschool children’s health and well-being is well-established [48]. However, time trends in pedometer-determined daily step counts in association with the part of the day (kindergarten and leisure-time), type of the day (weekdays and weekends), or the level of body weight (non-obese and obese) have not yet been sufficiently assessed. Unlike previous studies [17, 20, 23], and in accordance with other recent study [22], we uncovered significantly more (*p* < 0.001) overall daily PA in preschool boys than girls. In addition, similarly to a sample of 7–9-year-old Swedish children between 2000 and 2006 [49], a significantly higher (*p* < 0.05) daily step count was also noted in Czech preschoolers in 2015 compared to 2005. Physical activity performed during time spent in the kindergarten demonstrably contributed to higher daily PA on weekdays in 2015 (Figs. 2 and 3).

A distinctive change in the proportion of kindergarten/leisure-time step count, compared to the total daily step count between 2005 and 2015, was found in all participating preschoolers. In this period, the decline of leisure-time PA on weekdays was compensated for by the increase in kindergarten PA in the entire sample. This finding supports the contention that the PA programme in public kindergartens successfully promotes PA in children, regardless of their gender, age, and/or body weight [32]. Unlike English children [50], Czech preschool children can spend a substantial part of the day (from 6:15 am to 15:30 pm) in public kindergarten, from Monday to Friday during work weeks (40 weeks in 2015), except for public holidays and summer school holidays. Public kindergartens in the Czech Republic provide cheap (subsidised by the state) education for all 4–7-year-old preschool children; the final year prior to entering primary school is completely free. In addition to the standard daily PA programme (at least a 30–60 min walk outdoors and one 20 min period of indoor exercise) in public kindergartens may provide un-paid additional PA during the time spent outside in the playground/garden or indoors in the kindergarten classes under the supervision of the teacher [32].

Given the various current PA recommendation based on daily step count for preschool children (e.g. 9,000 steps/day [51]; or 11,500 steps/day [22, 32]) it is difficult to compare the step counts of preschool children across countries, races or even continents. However, the pedometer-determined daily step count of Czech 4–7-year-old preschool children in 2015 corresponds to the daily step count of Canadian boys (12,420 steps/day) and girls (11,438 steps/day) aged 5–7 years [19]. Nevertheless, a greater proportion of Czech preschoolers meet the recommended target of 11,500 steps/day [22, 32] on weekdays (45.4 %) and at weekends (45.9 %) than preschoolers from Belgium (40.0 % _weekdays_ and 20.5 % _weekends_), Bulgaria (29.3 % _weekdays_ and 29.2 % _weekends_), Greece (26.5 % _weekdays_ and 20.3 % _weekends_) and Poland (43.2 % _weekdays_ and 41.8 % _weekends_) [21]. At weekends, the percentage of Czech preschoolers meeting the 11,500 steps/day recommendation (>37.6 %_♀_ and >41.3 %_♂_) even surpasses the percentage of those from Germany (31.4 %) and Spain (37.0 %) [21].

In accordance with other recent studies [9, 14, 15] a relatively stable state (a non-significant increase) in the prevalence of obesity in Czech preschool children was observed between 2005 and 2015. The prevalence of obesity in Czech preschoolers in 2015 (9.52 % _BOYS_ and 9.20 %_ GIRLS_) remains lower than the current prevalence of obesity in New Zealand preschoolers aged 4–5 (18.7 %_ BOYS_ and 13.8 % _GIRLS_ [15]), English aged 2–5 (>13 %_ BOYS_ and >12 % _GIRLS_ [9]), and Chinese preschool boys aged 3–6 (13.3 % [14]). It is comparable to the prevalence of obesity among Scottish 6-year-old children (9 % [52]) and higher than in 5–6-year-old Italian preschool children (4.4 % [53]). Further comparisons of obesity prevalence among preschool children in the different studies are, however, hindered by usage of different standards for obesity.

### The strengths and limitations of the study

#### The strengths

This study employed the same research design in 2005 and in 2015. The use of identical pedometers, the same measurements and procedures, as well as trained research leaders in both data collection waves, can be considered as strengths. We included only data from children whose PA was monitored for at least eight hours a day, on at least four weekdays and both weekend days a week. These inclusion criteria regarding the length of the daily PA monitoring and the number of valid monitored days of week are more stringent than in previous studies [21, 49, 54] and therefore enhance the credibility of step count comparisons between weekdays and weekend days (or between kindergarten and leisure-time).

#### Limitations

Several limitations to our study need to be mentioned. Pedometers provide an inexpensive objective assessment of a child’s day-long PA [55] or PA during a certain parts of the school day [54, 56] and free-living activities [36, 37] where only a measure of total amount of PA and not the intensity or pattern of PA is required [37, 57]. These issues may affect the validity of the information gathered from young children, given the spontaneous, highly transitory, and intermittent pattern of their PA regardless of intensity, with most PA action lasting between 3 and 22 s [58]. Using an accelerometer with a short time sampling interval (epoch length e.g., 1, 2 or 5 s) of accelerometer counts could have added more information about the intensity of PA, time spent in moderate-to-vigorous PA, or sedentary time [57–59], but controversies exist regarding accelerometer epoch length as well as cut-off points of sedentary, light, and moderate-to-vigorous intensity activity in preschool children [60–62]. The absence of information about leisure-time activities of preschool children can be considered as a further limitation of the study. There were a relatively small number of participants and the public kindergartens were selected non-randomly. The stratified selection of public kindergartens and sample size require cautious generalisation of our results to the wider population of preschool children in the Czech Republic.

## Conclusion

Uncovering the trends of objectively measured PA, as well as the level of body weight, provides deeper insights into kindergarten and leisure-time PA behaviour and can serve as evidence in development of effective interventions aimed at increasing PA and subsequently reducing the incidence of childhood obesity. The level of pedometer-determined PA of girls and boys was higher in 2015 than in 2005. The higher level of PA in 2015 than in 2005 resulted from the increased PA in kindergarten time. Thus, the current PA program in public kindergartens effectively compensates for the decline in PA in leisure-time of weekdays, and even helps to increase the week-long PA of non-obese and obese preschoolers of both genders compared to 2005 and 2015. The prevalence of obesity among preschool girls and boys remained stable (<10 %) between 2015 and 2005.

## Abbreviations

BMI, Body Mass Index; Fisher’s LSD test, Fisher’s Least Significant Difference test; PA, Physical activity; SPSS, Statistical Package for the Social Sciences; WHO, World Health Organization.
